# Neuroprotective Effects of Dehydroepiandrosterone Sulphate Against Aβ Toxicity and Accumulation in Cellular and Animal Model of Alzheimer’s Disease

**DOI:** 10.3390/biomedicines13020432

**Published:** 2025-02-11

**Authors:** Barbara Vuic, Tina Milos, Erika Kvak, Marcela Konjevod, Lucija Tudor, Szidónia Farkas, Gordana Nedic Erjavec, Matea Nikolac Perkovic, Dora Zelena, Dubravka Svob Strac

**Affiliations:** 1 Laboratory for Molecular Neuropsychiatry, Ruder Boskovic Institute, Division of Molecular Medicine, Bijenicka cesta 54, 10 000 Zagreb, Croatia; barbara.vuic@irb.hr (B.V.); tina.milos@irb.hr (T.M.); marcela.konjevod@irb.hr (M.K.); lucija.tudor@irb.hr (L.T.); gordana.nedic.erjavec@irb.hr (G.N.E.); mnikolac@irb.hr (M.N.P.); 2 Laboratory of Behavioural and Stress Studies, Institute of Physiology, Medical School, University of Pécs, 7624 Pécs, Hungary; erika.kvak@aok.pte.hu (E.K.); szidonia.farkas@aok.pte.hu (S.F.); dora.zelena@aok.pte.hu (D.Z.)

**Keywords:** Aβ oligomers, Alzheimer’s disease, dehydroepiandrosterone sulphate, neuroprotection, primary neurons, 3xTg-AD mice

## Abstract

**Background/Objectives:** Beneficial effects of neurosteroid dehydroepiandrosterone sulphate (DHEAS) on cognition, emotions and behavior have been previously reported, suggesting its potential in the prevention and treatment of various neuropsychiatric and neurodegenerative disorders, including Alzheimer’s disease (AD). This study aimed to investigate the potential neuroprotective actions of DHEAS against Aβ toxicity in both cellular and animal models of AD. **Methods:** After optimizing the AD model in vitro, we investigated the DHEAS effects on the viability and death of primary mouse neurons exposed to toxic Aβ_42_ oligomers for 24 h. In order to extend our research to an in vivo study, we further tested the acute effects of intraperitoneal DHEAS administration on the Aβ plaque density in different brain regions of 3xTg-AD mice, an animal model of AD. **Results:** In cell culture, DHEAS hampered the decrease in the neuronal viability caused by toxic Aβ oligomers, primarily by influencing mitochondrial function and apoptosis. DHEAS also counteracted the increase in the mRNA expression of selected genes (*PI3K, Akt, Bcl2, Bax*), induced in neuronal culture by treatment with Aβ_42_ oligomers. Obtained data suggested the involvement of mitochondria, caspases 3 and 7, as well as the PI3K/Akt and Bcl2 signaling network in the antiapoptotic properties of DHEAS in neurons. Forty-eight hours after DHEAS treatment, a significantly lower number of Aβ plaques was observed in the motor cortex but not in other brain areas of 3xTg-AD mice. **Conclusions:** Results indicated potential neuroprotective effects of DHEAS against Aβ toxicity and accumulation, suggesting that DHEAS supplementation should be further studied as a novel option for AD prevention and/or treatment.

## 1. Introduction

Alzheimer’s disease (AD), the most common form of dementia, is a progressive neurodegenerative disorder that primarily affects neurons in the cortex and hippocampus [1]. Neuroimaging in AD patients revealed reduced brain volume and weight, along with enlarged lateral ventricles and sulci, while post-mortem brain studies identified extracellular amyloid-beta (Aβ) plaques, as well as intracellular neurofibrillary tangles resulting from hyperphosphorylated tau protein accumulation [2]. In AD, the enzymatic cleavage of amyloid precursor protein (APP) produces Aβ_40_ and Aβ_42_ peptides, which gradually form oligomers and amyloid plaques, with Aβ_42_ oligomers considered particularly neurotoxic [3]. Aβ dysregulation has been proposed to contribute to oxidative stress, mitochondrial dysfunction, disruption of membrane integrity, astrocyte and microglia activation, conversion of tau protein into insoluble tangles, as well as impaired axonal transport and synaptic transmission, leading to the damage and death of neurons [4]. Acetylcholinesterase inhibitors, donepezil, rivastigmine and galantamine, as well as the N-methyl-D-aspartate (NMDA) receptor antagonist, memantine, are only able to alleviate AD symptoms [5]. Two anti-amyloid monoclonal antibodies, lecanemab and aducanumab, represent the first disease-modifying drugs recently approved for AD treatment in the US [6]. However, since they have significant adverse effects, the most severe of which are amyloid-related imaging abnormalities (ARIAs) that can cause both brain edema (ARIA-E) and hemorrhage (ARIA-H), and as they demonstrate limited efficacy, primarily in early or middle stages of AD, further search for effective preventive/therapeutic approaches continues [7,8].

Dehydroepiandrosterone (DHEA) and its sulphate (DHEAS), together often referred to as DHEA(S), are among the most abundant endogenous steroid hormones in humans, secreted mainly by the adrenal cortex and gonads [9], with DHEAS representing the more stable and major circulating form [10]. However, since they are also produced de novo in the brain, where DHEA(S) concentrations are six to eight times higher than in peripheral blood, they were designated as neurosteroids [11,12]. DHEA(S) levels peak in the mid-20s and afterward steadily decline with age, resulting in only 10–20% of their maximum levels by 60–80 years of age [13] and coinciding with an increase in different age-related illnesses, including AD [14,15]. Previous research demonstrated beneficial effects of DHEA(S) on cognition, emotions and behavior [16,17], possibly via their actions on neurogenesis, neurite growth, neuroprotection, neurotransmitter systems, apoptosis, as well as through anti-inflammatory, anti-oxidant, and anti-glucocorticoid effects [18,19,20]. DHEA(S) has been suggested to directly and indirectly activate various receptors [20,21] and affect neuronal survival, at least partly, via modulation of phosphatidyl-inositol 3-kinase (PI3K)/Akt and NF-κB signaling pathways, which influence the transcription of Bcl-2 family proteins, important regulators of apoptosis [22,23,24]. Since various studies demonstrated that DHEA(S) can cross the blood–brain barrier [25,26], DHEA(S) supplementation shows potential in the prevention and treatment of various neuropsychiatric and neurodegenerative disorders [27,28]. However, due to contradictory findings, further studies are needed to investigate the therapeutic benefits of DHEA(S) in AD [29,30]. Specifically, in both in vitro and in vivo studies, timing of administration, dose administered, as well as various influences such as neurotoxins, hormones, or physiological state of the organism, have been shown as important factors affecting whether DHEA(S) is neuroprotective, ineffective or even neurotoxic [19]. In addition, it has been demonstrated that DHEA and DHEAS may have different effects and act through distinct mechanisms [24,31,32]. Finally, despite encouraging preclinical findings, limited clinical trials found that the beneficial cognitive effects of DHEA(S) in AD patients are mild if present at all [18].

Since elucidating Aβ’s neurotoxic mechanisms is crucial for understanding various brain dysfunctions of AD patients and Aβ-targeted drug development, in our study we focused on investigating the potential neuroprotective effects of DHEAS against Aβ toxicity and accumulation in both cellular and animal models of AD. Most of the previous in vitro studies that addressed the neuroprotective effects of DHEA(S) against Aβ-toxicity used neuronal cell lines and/or treatment with Aβ_42_ peptides that are known to form neurotoxic aggregates [33,34,35]. On the other hand, we have chosen primary mouse neurons, due to the fact that immortalized cell lines or those derived from tumors differ both genetically and phenotypically from normal, differentiated neurons originating from the brain. Specifically, various studies demonstrated that neuronal cell lines might lack the expression of some functional receptors, ion channels, enzymes or mature neural markers, which can also potentially influence their sensitivity to neurotoxins such as Aβ [36,37,38]. Therefore, although neuronal cell lines may be easier to work with and demonstrate a fast growth rate, primary neuronal culture, which retains morphological, neurochemical, and electrophysiological properties of neurons in vivo, is advantageous in its predictive capacity for drug research and development. Moreover, to assess DHEAS neuroprotective properties, in contrast to most previous in vitro studies, we have treated primary neurons with Aβ_42_ oligomers, shown to be more toxic than monomers and mature fibrils [39,40,41,42,43].

In addition, in order to confirm our findings in vivo, we have investigated the effects of DHEAS administration on the Aβ plaque density in the brain of 3xTg-AD triple transgenic mice (B6;129-Tg(APPSwe,tauP301L)1LfaPsen1tm1Mpm/Mmjax), homozygous for three human-based genetic mutations (*PSEN1, APPSwe, tauP30IL*) associated with AD. 3xTg-AD mice represent the widely used animal model of AD and are superior compared to rodent models in which Aβ is delivered into the brain by microinjections or infusions [44] since it replicates major histopathological and behavioral AD hallmarks, including progressive development of Aβ plaques with age [45,46,47]. Studies that assessed the neuroprotective actions of DHEAS against Aβ toxicity by using both in vitro and in vivo models of AD in parallel are rare [34,35]. Specifically, to study DHEAS neuroprotective effects, El Bitar et al. (2014) used Aβ_25–35_-treated B104 neuroblastoma cells, as well as male Swiss mice injected intracerebroventricularly with Aβ_25–35_ peptides [34], whereas Lin et al. (2024) used a combination of Aβ_1–42_-treated N2A mouse neuroblastoma cells and transgenic 5XFAD mice as the animal AD model [35]. As far as we know, this is the first study investigating DHEAS neuroprotective properties by simultaneously using primary neuronal culture and transgenic animals as optimal systems to model Aβ-induced neurotoxicity in AD pathology.

## 2. Materials and Methods

### 2.1. Cellular Model of AD

#### 2.1.1. Primary Neuronal Culture

Primary neuronal culture was obtained from 15.5-day-old C57BL/6 mouse embryos, according to the adapted protocol of Hilgenberg and Smith [48]. Pregnant mouse females were euthanized by cervical dislocation, the embryos were removed from amniotic sacs, decapitated, and their brains extracted and dissected under a microscope. Olfactory bulbs, cerebellum and meninges were removed, and neurons from remaining brain tissue were incubated with 0.125% trypsin (Sigma-Aldrich, St. Louis, MO, USA) in Hank’s balanced salt solution (HBSS, Lonza, Portsmouth, NH, USA), followed by the addition of 10 µg/mL of DNase I solution (Sigma-Aldrich, St. Louis, MO, USA). Neurons were further dispersed by gentle trituration through a sterile glass Pasteur pipette and passed through a 70 μm cell strainer. Cell suspension was centrifuged at 1000 rpm for 5 min, and the pellet was resuspended in Dulbecco’s modified Eagle’s medium (DMEM-high glucose, Sigma-Aldrich, St. Louis, MO, USA) with 10% fetal bovine serum (Gibco, Grand Island, NY, USA), and 2 mM L-glutamine (Sigma-Aldrich, St. Louis, MO, USA). Cells were counted using LUNA-II^TM^ Automated cell counter (Logos Biosystems, Annandale, VA, USA) and seeded on the poly-D-lysine (Sigma-Aldrich, St. Louis, MO, USA) coated plates at a density of 1.5 × 10^5^ cells/well (12-well plates) or 1.5 × 10^4^ cells/well (96-well plates). After 4 h, DMEM was replaced with Neurobasal medium (Thermo Fisher Scientific, Waltham, MA, USA) with 2% B-27 supplement (Thermo Fisher Scientific, Waltham, MA, USA) and 0.5 mM L-glutamine (Sigma-Aldrich, St. Louis, MO, USA), and neuronal culture was maintained at 37 °C and 5% CO_2_. Every 3 days, one-half of the neurobasal medium was replaced with fresh medium. Five to seven days after seeding, neurons were treated and used for analyses.

For confirmation of neuronal culture by immunocytochemistry (Supplementary Figure S1), neurons were grown on poly-D-lysine (Sigma-Aldrich, St. Louis, MO, USA) coated coverslips for 5–7 days and then fixed and permeabilized by incubation with cold 100% methanol. After washing, non-specific binding was blocked with blocking buffer (0.1% PBS-Tween with 1% BSA, 10% goat serum, 0.3 M glycine) for 1 h at 37 °C. Cells were then incubated with primary Anti-beta III Tubulin (neuronal marker, Abcam, Cambridge, UK) antibody (diluted in 1% BSA buffer 1:1000) overnight at 4 °C, washed, and incubated with Goat Anti-Rabbit IgG H&L (Alexa Fluor 488) (Abcam, Cambridge, UK) antibody (diluted in 1% BSA buffer 1:1000) for 1 h at RT. After washing, nuclei were stained with 1 μg/mL Hoechst 33342 (Sigma-Aldrich, St. Louis, MO, USA) for 1 min and washed again. The cells were mounted (Fluoromount, Sigma-Aldrich, St. Louis, MO, USA) and visualized using the Olympus BX51 Fluorescence Microscope (Olympus, Tokio, Japan).

#### 2.1.2. Preparation and Characterization of Aβ_42_ Monomers, Oligomers and Polymers

Monomers, oligomers, and polymers were prepared from synthetic human amyloid-β (1–42) peptide (Aβ_42_, California Peptide Research, Napa, NC, USA), as described by Stine et al. [49]. Briefly, Aβ_42_ peptide was dissolved to 1 mM in hexafluoroisopropanol (HFIP, Sigma-Aldrich, St. Louis, MO, USA) and separated into 10 μL aliquots. HFIP was removed under vacuum in a SpeedVac (Thermo Fisher Scientific, Waltham, MA, USA). Obtained Aβ_42_ peptide film was dissolved in 2 μL dimethyl sulfoxide (DMSO, Sigma-Aldrich, St. Louis, MO, USA), mixed, centrifuged and sonicated. For oligomeric conditions, ice-cold DMEM/F-12 culture media (Sigma-Aldrich, St. Louis, MO, USA) is added to a final concentration of 100 μM, mixed and incubated at 4 °C for 24 h. To obtain 100 μM polymers (fibrils), 10 mM HCl is added, mixed and incubated for 24 h at 37 °C, whereas for 100 μM monomers, phosphate-buffered saline (PBS) was added, mixed and used immediately.

Preparations of Aβ_42_ monomers, oligomers and polymers in 10 µM concentration were verified by multimode atomic force microscopy (AFM) (Supplementary Figure S2). Briefly, samples were prepared by spotting 10 μL of solution on freshly cleaved mica, allowed to settle and attach to the surface for 45 min, rinsed twice with 50 μL ultrapure water, and excess water was allowed to evaporate [50,51]. Topography images were acquired using a Nanoscope IIIa controller (Bruker, Bremen, Germany) with a vertical engagement (JV) 125 μm scanner. Tapping mode imaging was performed using silicon tips (RTESP, resonance frequency 289–335 kHz, spring constant in 20–80 N/m range; Bruker). The set point amplitude to free amplitude ratio (A/A0) was maintained at 0.9 (light tapping). A linear scanning rate was optimized between 1.0 and 2.0 Hz, with a scan resolution of 512 samples per line. Images were analyzed using NanoScopeTM software (Digital Instruments, V6.14r1, Bruker, Bremen, Germany) and presented as raw data, except for first-order two-dimensional flattening. All measurements were performed in air at RT and 50–60% relative humidity.

Additionally, the preparation of Aβ_42_ monomers, oligomers, and polymers was verified using vertical denaturing polyacrylamide gel electrophoresis (SDS-PAGE). Briefly, preparations of Aβ_42_ monomers, oligomers or polymers mixed with Laemmli sample buffer (Bio-Rad Laboratories, Hercules, CA, USA) at a 1:1 ratio in a total volume of 30 μL, along with 5 μL of Novex Sharp Pre-stained Protein Standard (Invitrogen, Carlsbad, CA, USA), were loaded onto a 1.5 mm tick 4–20% Mini-PROTEAN TGX Precast Gel (Bio-Rad Laboratories, Hercules, CA, USA). SDS-PAGE was performed in MES SDS running buffer (Invitrogen, Carlsbad, CA, USA) using Mini-PROTEAN III Cell system (Bio-Rad Laboratories, Hercules, CA, USA) at 100 V/80 mA for 90 min. Aβ_42_ monomers, oligomers and polymers in gel were visualized by Coomassie Brilliant Blue staining (Thermo Fisher Scientific, Waltham, MA, USA) (Supplementary Figure S2) at 50 °C for 8–10 min, followed by destaining.

#### 2.1.3. Cell Treatment and Determination of Viability, Cytotoxicity and Apoptosis

Five to seven days after seeding (37 °C, 5% CO_2_), primary neurons were used to test the toxic effects of various concentrations (0.1, 1, and 10 μM) of Aβ_42_ monomers, oligomers and polymers for 24 h. Moreover, preparations of Aβ_42_ oligomers were added to cell medium of primary neuronal culture at concentrations of 0.1, 1, and 10 μM for 24, 48, and 72 h to assess their toxicity. Finally, primary neurons were co-treated for 24 h with 10 µM Aβ_42_ oligomers in medium and different concentrations (10^−8^ to 10^−6^ M) of DHEAS (Supelco, Bellefonte, PA, USA) diluted in milli-Q water. Neurobasal medium was removed from cells and replaced with fresh medium containing 10 µM Aβ_42_ oligomers and different concentrations of DHEAS or vehicle.

Colorimetric MTT assay was performed in 96-well plates to evaluate cell viability. Briefly, after the removal of thef culture medium, 40 μL of 0.5 mg/mL MTT solution (Sigma-Aldrich, St. Louis, MO, USA) diluted in neurobasal medium was added to each well, and plates were incubated in the dark at 37 °C and 5% CO_2_. Following 4 h of incubation, formed purple formazan crystals were dissolved by adding 160 μL of DMSO (Sigma-Aldrich, St. Louis, MO, USA) to each well and shaking. Absorbance was measured at 570 nm using the Thermo Labsystems Multiskan EX Microplate Reader (Thermo Fisher Scientific, Waltham, MA, USA). Since only viable cells have active metabolism and can convert MTT to formazan, the amount of produced formazan, measured spectrophotometrically is proportional to the number of viable cells. Total cell count and viability, as well as activation of the Bcl-2 pathway, were analyzed using the MUSE Count & Viability Assay Kit (Luminex, Austin, TX, USA) and the MUSE Bcl-2 Activation Dual Detection Assay Kit (Luminex, Austin, TX, USA) on the Guava MUSE™ Cell Analyzer (Luminex, Austin, TX, USA). In the MUSE Count & Viability Assay Kit, both viable and non-viable cells are differentially stained based on their permeability to the DNA-binding dyes in the reagent, whereas MUSE Bcl-2 Activation Dual Detection Assay Kit uses a pair of antibodies; with one antibody detecting total Bcl-2 protein expression and the other detecting the phosphorylated form of Bcl-2.

Additionally, RealTime-Glo™ MT Cell Viability Assay (Promega, Madison, WI, USA) was used to determine the number of viable cells in the culture. Since only live metabolically active cells can reduce the substrate to form the NanoLuc^®^ Substrate, used rapidly by NanoLuc^®^ Luciferase, light production is proportional to the number of live cells in culture. Mitochondrial ToxGlo™ Assay (Promega, Madison, WI, USA) was applied to assess cellular ATP levels and cell membrane integrity. Cell membrane integrity is first assessed by measuring the activity of proteases released from membrane-compromised cells using a fluorogenic peptide substrate bis-AAF-R110, which cannot cross intact membranes of live cells and is used to assess protease activity associated with necrosis. To assess potential mitochondrial dysfunction, ATP is measured by adding ATP detection reagent, resulting in cell lysis and generation of a luminescent signal proportional to the amount of ATP. Caspase-Glo 3/7 Assay (Promega, Madison, WI, USA) was used to measure the activities of caspases 3 and 7, as critical mediators of apoptosis. Adding Caspase-Glo 3/7 reagent results in cell lysis, followed by caspase-3/7 cleavage of proluminescent DEVD-aminoluciferin substrate. Free aminoluciferin is consumed by thermostable luciferase, generating a luminescent signal, proportional to caspase-3/7 activity. All fluorescence/luminescence measurements were conducted using Infinite 200 PRO microplate reader (Tecan, Männedorf, Switzerland) according to the manufacturer’s instructions.

To distinguish between apoptosis and necrosis, Hoechst 33342 and propidium iodide (PI) staining, respectively, were applied to neurons in 12-well plates. A total of 1 µL of Hoechst 33342 (Sigma-Aldrich, St. Louis, MO, USA) and 1 µL of PI (Sigma-Aldrich, St. Louis, MO, USA) were added to each well and incubated for 3 min at RT in the dark. Apoptotic and necrotic cells were visualized using a blue filter for Hoechst 33342 (357 nmEx, 447 nmEm) and a red filter for PI (531 nmEx, 593 nmEm) on an EVOS Cell Imaging Station (Thermo Fisher Scientific, Waltham, MA, USA). Neurons were counted using ImageJ/Fiji software for Windows bundled with 64-bit Java 8. (https://imagej.net/ij/download.html, accessed on 14 January 2025; National Institutes of Health, Bethesda, MA, USA), and proportions of apoptotic and necrotic cells relative to the total cell number were calculated.

#### 2.1.4. RNA Expression Analysis

To isolate RNA from neurons, neurobasal medium was removed from 6-well plates and cells were washed twice with 500 µL of PBS. Neurons were detached by 2–3 min incubation with 100 µL of 0.5% trypsin. After adding 900 µL of fresh medium, cells were collected into a sterile 1.5 mL tube and centrifuged at 2000× *g* for 5 min at 4 °C. Total RNA was extracted using the PureLink RNA Mini Kit (Thermo Fisher Scientific, Waltham, MA, USA) following the manufacturer’s instructions. Briefly, after discarding the supernatant, 0.3 mL of lysis buffer with 2-mercaptoethanol was added to the cell precipitate and vortexed. Lysate was resuspended 5–6 times through an 18–21-gauge injection needle attached to a syringe. A 1.5 volume of 70% ethanol was added and vortexed, and 700 µL of cell lysate was transferred to spin cartridge (with collection tube), centrifuged at 12,000× *g* for 15 s at RT and flow-through was discarded. This step was repeated 3–4 times for the rest of the sample. A total of 700 µL of wash buffer 1 was added to the spin cartridge, centrifuged at 12,000× *g* for 15 s at RT, and flow-through was discarded. In total, 500 μL of wash buffer 2 with ethanol was added to the spin cartridge, centrifuged at 12,000× *g* for 15 s at RT, and flow-through was discarded. This step was repeated once more. After centrifugation at 12,000× *g* for 1 min at RT, the spin cartridge was inserted into the recovery tube, 100 μL RNase-free water was added, incubated 1 min at RT and centrifuged at 12,000× *g* for 2 min at RT. Purity and quantity of eluted RNA were determined at 260/280 nm using NanoPhotometer N60 (Implen, München, Germany).

Expression of *PI3K, Akt, Bcl2,* and *Bax* genes was determined by real-time PCR on a Real-time PCR ABI PRISM 7300 SDS (Applied Biosystems, Waltham, MA, USA) using qPCRBIO SyGreen 1-Step Detect Hi-ROX kit (PCR Biosystems, London, UK). Amplification forward (Fw) and reverse (Rv) primers were: *PI3K* Fw 5′-CAGTTTGGTGTCATCCTGGAAGC-3′ and Rv 5′-TCTGCTCAGCTTCACCGCATTC-3′, *Akt* Fw 5′-GGACTACTTGCACTCCGAGAAG-3′ and Rv 5′-CATAGTGGCACCGTCCTTGATC-3′, *Bcl2* Fw 5′-CCTGTGGATGACTGAGTACCTG-3′ and Rv 5′-AGCCAGGAGAAATCAAACAGAGG-3′, and *Bax* Fw 5′-CCGGCGAATTGGAGATGAA-CTG-3′ and Rv 5′-AGCTGCCACCCGGAAGAAGACCT-3′. Final reaction volume was 20 μL per well (10 μL of 2× qPCRBIO SyGreen 1-Step Mix, 0.8 µL of 10 µM forward primer, 0.8 µL of 10 µM reverse primer, 1 µL of 20× RTase Go, 10 ng total RNA, up to 20 µL PCR grade dH_2_O). PCR conditions involved: initial hold (42 °C, 2 min), reverse transcription (45 °C, 10 min), polymerase activation (95 °C, 2 min), then 40 cycles of denaturation (95 °C, 5 s), and annealing/extension (65 °C, 30 s), with final dissociation step (95 °C (15 s), 60 °C (1 min), 95 °C (15 s) and 60 °C (15 s)). Relative mRNA quantification for selected genes was conducted using the glyceraldehyde-3-phosphate dehydrogenase (GAPDH) as the reference gene, employing the 2^−ΔΔCt^ method.

### 2.2. Animal Model of AD

#### 2.2.1. 3xTg-AD Mice

This study enrolled 6-month-old male transgenic 3xTg-AD mice (weighing 25–30 g), bred and housed at the Institute of Physiology, Medical School, University of Pécs, under controlled conditions (12-h light/dark cycle, temperature 22 ± 2 °C, relative humidity 55 ± 10%, and ad libitum access to food and water). Mice were conventionally caged in groups of six. All experimental procedures were conducted according to the guidelines of the Animal Welfare Committee of the University of Pécs (BA02/2000-84/2022) and European Parliament and Council Directive (2010/63/EU) on the protection of animals used for scientific purposes. Procedures performed on animals were carried out in compliance with the 3R principle. The persons who handled the animals were officially trained to work with laboratory animals.

#### 2.2.2. DHEAS Treatment and Brain Tissue Processing

At 6 months of age, 3xTg-AD mice were divided into control (vehicle: sterile water) and DHEAS (10 mg/kg DHEAS dissolved in sterile water) groups. A single intraperitoneal (i.p.) treatment was administered after weighing the animals. Subsequently, as shown in a graphical timeline, animals were categorized into four groups based on the time of sacrifice: control 24 h (n = 6), DHEAS 24 h (n = 6), control 48 h (n = 6), and DHEAS 48 h (n = 6).

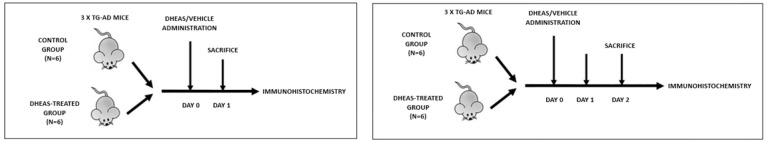



Graphical timeline of animal study design.

Mice were anesthetized by i.p administration of 10 mL/kg ketamine-xylazine (125 mg/kg ketamine and 25 mg/kg xylazine in 0.9% sodium-chloride solution), 24 h or 48 h following treatment and then perfused with 15 mL of 0.9% sodium-chloride solution followed by 30 mL of 4% paraformaldehyde (PFA). Brains were dissected, post-fixed in 4% PFA for 3 h, and dehydrated in 30% sucrose solution overnight. Brain slices of 30 µm thickness were prepared using a Leica SM2010 R Sliding Microtome (Leica Microsystems, Wetzlar, Germany) for immunohistochemical staining.

#### 2.2.3. Immunohistochemistry

To investigate Aβ plaques in mice brains, peroxidase-based immunohistochemistry with nickel-diaminobenzidine tetrahydrochloride (Ni-DAB) visualization was employed [52]. Each brain slice underwent pre-treatment, including a 3-min exposure to 100% formic acid (Sigma-Aldrich, St. Louis, MO, USA) to enhance epitope accessibility, followed by peroxidase blocking (1% peroxide solution, 15 min) and background permeabilization (0.2% triton-X, 10% horse serum in 0.05 Tris solution, 2 h). Primary antibody incubation against Aβ (Rabbit, 1:500, Invitrogen, Carlsbad, CA, USA) was conducted in 0.02% triton-X, 5% horse serum, and Tris solution for 72 h. Consequently, samples underwent biotinylated secondary antibody incubation (biotinylated anti-rabbit, 1:200, Jackson ImmunoResearch, West Grove, PA, USA) for 2 h, followed by visualization using a VECTASTAIN Elite ABC-Peroxidase Kit (Vector Laboratories, Newark, CA, USA), Ni-DAB (Sigma-Aldrich, St. Louis, MO, USA) and glucose oxidase (Sigma-Aldrich, St. Louis, MO, USA) solution. Imaging was performed using a Nikon Eclipse E1 R microscope (Nikon, Tokyo, Japan) at 4× magnification. Aβ_43_ plaques were quantified using ImageJ/Fiji, analyzing at least 3 slices per animal per brain region. Regions of interest included the motor cortex (MC, between bregma +2.93 and +1.77 mm), the somatosensory cortex (SSC, between bregma +0.50 and −1.20 mm), the subiculum region of the hippocampus (HC, between bregma −2.45 and −3.07 mm), and the basolateral amygdala (BLA, between bregma −0.83 and −1.55 mm) [53]. To mitigate potential false results due to time variables, the number of Aβ plaques in DHEAS groups was normalized against those in control groups after each staining. Different control means were also used for 24 h and 48 h groups to account for variations in perfusion.

### 2.3. Statistical Analysis

Statistical analysis was performed using GraphPad Prism version 4.00 for Windows (GraphPad Software, Inc., San Diego, CA, USA). In the cellular AD model, each experiment included at least three replicate measurements, while in the animal AD model, results were obtained from six mice per group. The D’Agostino and Pearson omnibus normality test was used to assess the normality of data. Data are presented as means ± standard deviations (SD) and compared using Student’s *t*-test (for comparison of 2 groups) or one-way ANOVA, followed by the post-hoc Tukey’s multiple comparison test (for comparison of 3 or more groups). A two-way ANOVA, followed by the post-hoc Tukey’s multiple comparison test was used for evaluation of the effects of two independent variables and their interaction. *p* < 0.05 was considered statistically significant.

## 3. Results

**Figure 1:**
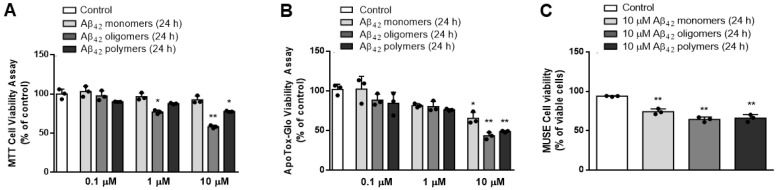
Treatment with various concentrations (0.1–10 μM) of Aβ_42_ monomers, oligomers and polymers for 24 h affected the viability of primary mouse neurons determined by (**A**) MTT Assay: * *p* < 0.005, ** *p* < 0.0001 vs. control; (**B**) RealTime-Glo™ MT Assay: * *p* < 0.002, ** *p* < 0.0001 vs. control; and demonstrated that 10 μM Aβ_42_ oligomers were most toxic, as confirmed with (**C**) MUSE Count and Viability reagent using MUSE™ cell analyzer: ** *p* < 0.0001 vs. control. Results are expressed as means ± SD from 3 experiments and analyzed by ANOVA followed by Tukey’s multiple comparisons test. Dots represent individual values.

To model AD in vitro, we first exposed primary mouse neurons to various concentrations (0.1–10 μM) of Aβ_42_ monomers, oligomers and polymers for 24 h. As demonstrated by an MTT viability test, Promega RealTime-Glo MT Cell Viability assay and MUSE Count and Viability Assay (Supplementary Figure S3), 10 μM Aβ_42_ oligomers administered for 24 h were the most toxic (*p* < 0.0001, ANOVA followed by Tukey’s test) to primary mouse neurons, when compared to Aβ_42_ monomers or polymers (Figure 1).

**Figure 2:**
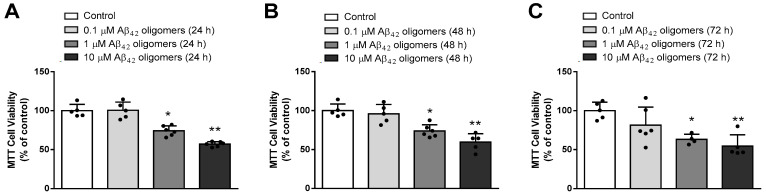
Treatment with various (0.1–10 μM) concentrations of Aβ_42_ oligomers for (**A**) 24 h (**B**) 48 h and (**C**) 72 h, affected the viability of primary mouse neurons determined by MTT assay: * *p* < 0.01, ** *p* < 0.001 vs. control, and demonstrated significant neurotoxicity already after 24 h treatment with Aβ_42_ oligomers. Results are expressed as means ± SD from 4 to 6 experiments and analyzed by ANOVA followed by Tukey’s multiple comparisons test. Dots represent individual values.

We further investigated the toxic effects of various (0.1–10 μM) concentrations of Aβ_42_ oligomers in primary neurons during different times of exposure (24–72 h). As shown in Figure 2, the MTT test revealed that 24 h treatment with 10 μM Aβ_42_ oligomers was sufficient to exert significant toxic effects (*p* < 0.001, ANOVA followed by Tukey’s test) to neurons, and prolongation of treatment to 48 or 72 h had not decreased their viability any further.

**Figure 3:**
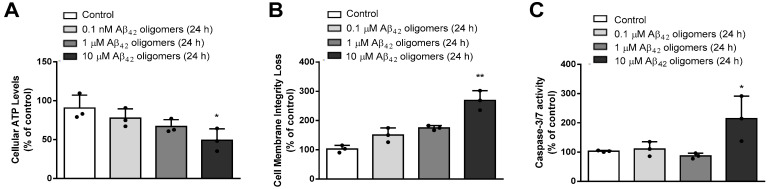
Treatment of primary mouse neurons with various (0.1–10 μM) concentrations of Aβ_42_ oligomers for 24 h affected (**A**) ATP levels: * *p* < 0.05 vs. control; (**B**) cell membrane integrity: ** *p* < 0.001 vs. control; (**C**) caspase 3/7 activity: * *p* < 0.02 vs. control; determined by Mitochondrial ToxGlo™ Assay and Caspase-Glo^®^ 3/7 Assay™. Results are expressed as means ± SD from 3 experiments and analyzed by ANOVA followed by Tukey’s multiple comparisons test. Dots represent individual values.

Therefore, primary neuronal culture treated with 10 μM Aβ_42_ oligomers for 24 h appeared as an optimal in vitro model of AD; however, in order to investigate this model in more detail, we conducted further studies. As shown in Figure 3, Promega Mitochondrial ToxGlo and Caspase-Glo 3/7 Assays demonstrated that administration of 10 μM Aβ_42_ oligomers to primary neurons for 24 h significantly decreased ATP levels (*p* < 0.05, ANOVA followed by Tukey’s test) and cell membrane integrity (*p* < 0.001, ANOVA followed by Tukey’s test), while increasing the activity of caspases 3 and 7 (*p* < 0.02, ANOVA followed by Tukey’s test), suggesting mitochondrial dysfunction leading to both cellular necrosis and apoptosis.

**Figure 4:**
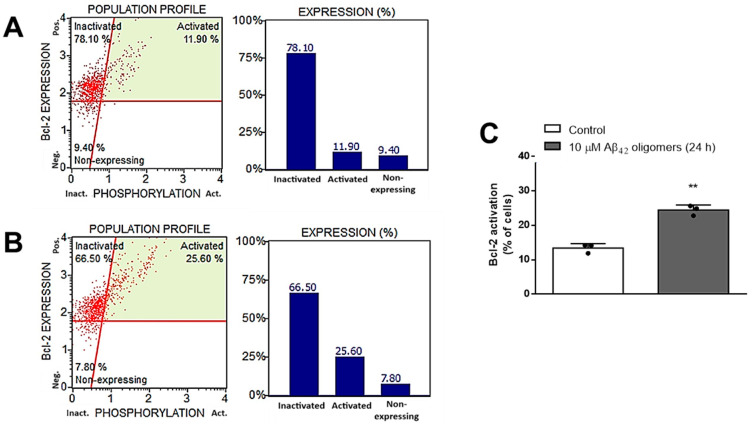
A representative sample of the cell Bcl-2 activation profile in primary mouse neurons treated for 24 h with (**A**) vehicle (control) (**B**) 10 μM Aβ_42_ oligomers, obtained by using MUSE Bcl-2 activation reagent and the MUSE™ cell analyzer, and (**C**) Bcl-2 activation levels expressed as means ± SD from 3 experiments and analyzed by the Student *t*-test: ** *p* < 0.001 vs. control. Dots represent individual values.

In line with these findings are results obtained by MUSE Bcl-2 Activation Dual Detection Assay Kit, which demonstrated that treatment with 10 μM Aβ_42_ oligomers for 24 h induced significant (*p* < 0.001, Student *t*-test) Bcl-2 activation (from 13.40 to 24.43%) in primary mouse neurons (Figure 4).

**Figure 5:**
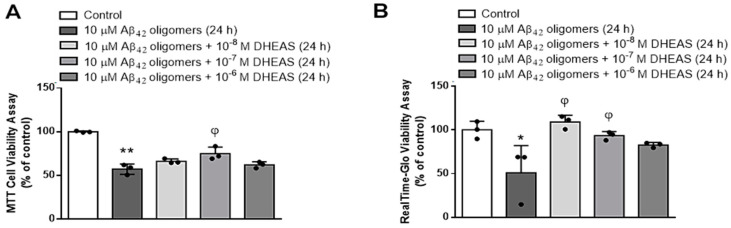
Effects of various DHEAS concentrations on the viability of primary mouse neurons treated with 10 μM Aβ_42_ oligomers for 24 h determined by (**A**) MTT Assay: ** *p* < 0.0001 vs. control, ^φ^ *p* < 0.01 vs. Aβ_42_ oligomers; (**B**) RealTime-Glo MT Assay: * *p* < 0.02 vs. control, ^φ^ *p* < 0.01 or *p* < 0.05 vs. Aβ_42_ oligomers. Results are expressed as means ± SD from 3 experiments and analyzed by ANOVA followed by Tukey’s multiple comparisons test. Dots represent individual values.

In order to investigate the potential neuroprotective properties of DHEAS in this in vitro AD model, we have examined the effects of various DHEAS concentrations (10^−6^–10^−8^ M) on the viability of primary mouse neurons treated with 10 μM Aβ_42_ oligomers for 24 h. As shown in Figure 5, MTT test and Promega RealTime-Glo MT Assay demonstrated that DHEAS simultaneously administered in the concentration of 10^−7^ M significantly (*p* < 0.01 or *p* < 0.05, respectively, ANOVA followed by Tukey’s test) counteracted the observed decrease in the neuronal viability, induced by toxic Aβ_42_ oligomers.

**Figure 6:**
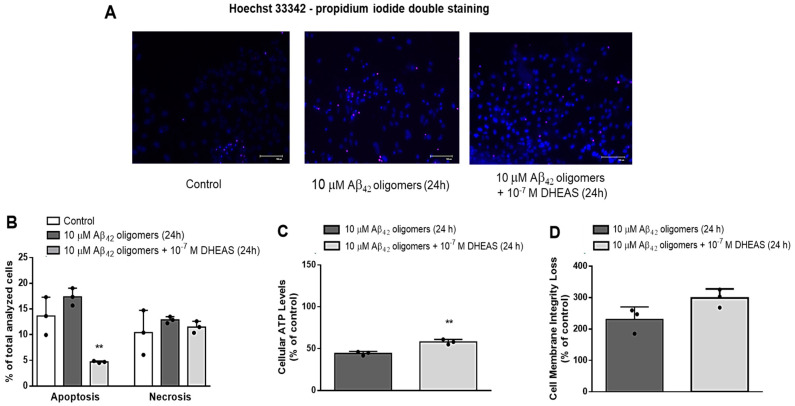
Effects of 10^−7^ M DHEAS on mitochondrial dysfunction, apoptosis and necrosis of primary mouse neurons treated with 10 μM Aβ_42_ oligomers for 24 h, determined by (**A**) Hoechst 33342 (blue) and propidium iodide (red) staining and EVOS system, Scale bar 100 μm; (**B**) ** *p* < 0.005 vs. Aβ_42_ oligomers, analyzed by two-way ANOVA followed by Tukey’s multiple comparisons test; and determined by Mitochondrial ToxGlo™ measuring (**C**) ATP levels: ** *p* < 0.003 vs. Aβ_42_ oligomers, analyzed by Student’s *t*-test and (**D**) cell membrane integrity. Results are expressed as means ± SD from 3 experiments. Dots represent individual values.

To distinguish the effects of 10^−7^ M DHEAS on apoptosis and necrosis of primary mouse neurons treated with 10 μM Aβ_42_ oligomers for 24 h, we stained the neurons with Hoechst 33342 and propidium iodide (PI) and visualized them using EVOS system (Supplementary Figure S4). Two-way ANOVA demonstrated significant effects of DHEAS treatment (*p* < 0.02) and significant interaction between DHEAS treatment and type of cell death (*p* < 0.035). Our findings revealed that 10^−7^ M DHEAS significantly (*p* < 0.005, Tukey’s test after two-way ANOVA) decreased the apoptosis but affected the necrosis of neurons to a much lesser extent, both induced by treatment with 10 μM Aβ_42_ oligomers for 24 h (Figure 6A,B). Moreover, Promega Mitochondrial ToxGlo Assay demonstrated that administration of 10^−7^ M DHEAS to primary neurons for 24 h significantly counteracted the decrease in cellular ATP levels (*p* < 0.003, Student *t*-test) (Figure 6C), but not cell membrane integrity loss (Figure 6D), both induced by 10 μM Aβ_42_ oligomers, suggesting DHEAS effects on mitochondrial function but not on the necrosis of neurons.

**Figure 7:**
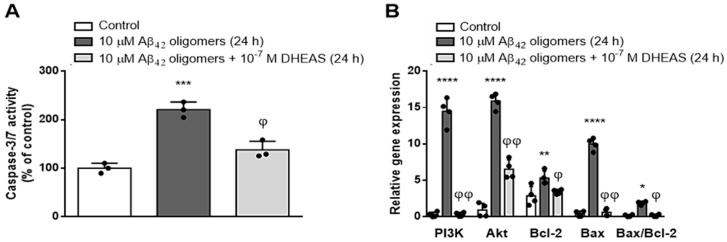
Effects of 10^−7^ M DHEAS administration in the primary mouse neuronal culture treated with 10 μM Aβ_42_ oligomers for 24 h on (**A**) caspase 3/7 activity determined with Caspase-Glo^®^ 3/7 Assay. *** *p* < 0.0005 vs. control, ^φ^ *p* < 0.004 vs. Aβ_42_ oligomers; (**B**) mRNA expression of selected genes (*PI3K, Akt, Bcl2, Bax, Bax/Bcl2* ratio) determined by qPCR and normalized to *GAPDH* expression. * *p* < 0.02, ** *p* < 0.001, **** *p* < 0.0001 vs. control; ^φ^ *p* < 0.002, ^φφ^ *p* < 0.0001 vs. Aβ_42_ oligomers. Results are expressed as means ± SD from 3–4 experiments and analyzed by one or two-way ANOVA followed by Tukey’s multiple comparisons test.

Since our results indicated that the observed neuroprotective effects of DHEAS against Aβ_42_ toxicity in primary neuronal culture are mainly due to the beneficial actions of DHEAS on mitochondrial function and against apoptosis, we further investigated the effects of 10^−7^ M DHEAS on the activity of caspases 3 and 7. As shown in Figure 7A, DHEAS administration significantly (*p* < 0.004, ANOVA followed by Tukey’s test) blocked the increase in the activity of caspases 3 and 7, induced in primary neurons by the toxic effects of 10 μM Aβ_42_ oligomers. In addition, we also investigated the mRNA expression of selected genes (*Bcl2, Bax, Akt, PI3K*), belonging to the signaling pathway involved in the regulation of apoptosis. Two-way ANOVA revealed a significant influence of treatment (*p* < 0.0001) and genes analyzed (*p* < 0.0001), as well as their interaction (*p* < 0.0001) on mRNA expression (Figure 7B). As demonstrated in Figure 7B, 10^−7^ M DHEAS significantly counteracted the increase in the mRNA expression of *PI3K, Akt, Bax* and *Bcl2* genes, as well as the *Bax/Bcl2* ratio, induced in neuronal culture by treatment with Aβ_42_ oligomers (Tukey’s test after two-way ANOVA). These findings together suggest the involvement of caspases 3 and 7, as well as PI3K/Akt and the Bcl2 signaling network in the antiapoptotic properties of DHEAS in neurons.

**Figure 8:**
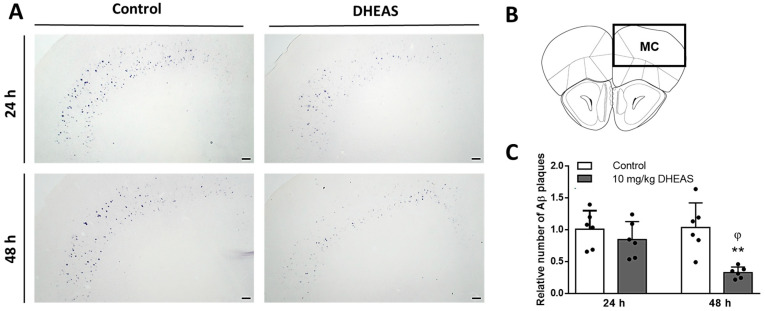
The effects of 10 mg/kg DHEAS on Aβ plaque density in the mouse motor cortex (MC) of 3xTg-AD mice 24 and 48 h after i.p. administration. (**A**) MC representative immunohistochemistry staining against Aβ, Scale bar 100 μm; (**B**) analyzed brain slices from MC; (**C**) relative number of Aβ plaques in the MC of 3xTg-AD mice 24 h and 48 h following vehicle (Control) or 10 mg/kg DHEAS i.p. administration: ** *p* < 0.002 vs. control, ^φ^ *p* < 0.03 vs. DHEAS 24 h group. Results are expressed as means ± SD from 6 mice per group and analyzed by two-way ANOVA followed by Tukey’s multiple comparisons test. Dots represent individual values.

Finally, in order to confirm our findings in vivo, we investigated the effects of DHEAS administration on the Aβ plaque density in different brain regions of 3xTg-AD mice, an animal model of AD. Results revealed varying numbers of Aβ_43_ plaques across different brain regions of 3xTg-AD mice, with the highest counts observed in the MC, followed by the BLA, HC, and SSC (Supplementary Table S1). Two-way ANOVA revealed that 10 mg/kg DHEAS treatment decreased the number of Aβ plaques in MC (*p* = 0.0012) of 3xTg-AD mice, in a time-dependent manner (*p* = 0.0465), as well as their significant interaction (*p* = 0.0289). There was a significant difference between 24 h and 48 h time points in the DHEAS-treated groups of mice (*p* = 0.0231 Tukey’s test after two-way ANOVA). As shown in Figure 8, 48 h after the treatment, DHEAS-treated 3xTg-AD mice had a significantly (*p* = 0.0017) lower number of Aβ plaques in MC compared to the control group (Tukey’s test after two-way ANOVA). However, in other investigated brain regions (SSC, BLA and HC), no changes in the Aβ plaque density between the groups were detected 24 or 48 h following DHEAS treatment (Supplementary Table S1).

## 4. Discussion

Many AD hypotheses implicating various mechanisms have been proposed; however, the amyloid hypothesis still represents the most accepted model of AD pathogenesis [54], offering an explanation for senile plaque formation in the brain, and is commonly used for investigation of novel diagnostic tools and therapeutic strategies [55]. Although Aβ_40_ and Aβ_42_ peptides are known for their significant propensity to form neurotoxic aggregates in the brain, longer Aβ isoforms, such as Aβ_43_, are also increasingly recognized for their heightened tendency to aggregate [56]. Initially, insoluble large Aβ fibril deposits were thought to be the main cause of neuronal damage [57]. However, more recent studies demonstrated that intermediate soluble Aβ oligomers are more toxic than both monomers and mature fibrils [39,40,41,42,43], whereas Aβ oligomers and fibrillar aggregates might activate different apoptotic pathways [43,58].

In line with these reports, our study also observed that the treatment of primary mouse neurons with Aβ_42_ oligomers exhibited the highest toxicity in comparison to monomers and polymers, especially when administered for 24 h in a 10 μM dose. Therefore, the treatment of primary neurons with 10 μM Aβ_42_ oligomers for 24 h was further evaluated as an in vitro model of AD. Our results demonstrated that administration of Aβ_42_ oligomers significantly decreased ATP levels and cell membrane integrity, while inducing Bcl-2 as well as caspase 3 and 7 activation, suggesting mitochondrial dysfunction leading to both cellular necrosis and apoptosis. Our findings support previous reports on mitochondrial dysfunction, as well as different types of neuronal cell death commonly observed in AD brain due to Aβ toxicity [59,60,61]. In contrast to necrosis, which is a form of traumatic cell death due to acute cellular injury, apoptosis is a highly regulated and controlled programmed cell death [62]. Various proapoptotic signals, including changes in the interplay between Bcl-2 family members [63,64], lead to abnormalities of mitochondrial function, such as the permeabilization of mitochondrial membrane and alteration in the inner membrane potential, decreased ATP production, and the release of Ca^2+^ ions [65]. Several factors released from mitochondria lead to DNA damage and the activation of initiator caspase 9 and executioner caspases 3 and 7 [66,67,68], which cleave cellular proteins, resulting in disruption of the plasma membrane and cell death. Similar to our findings, other studies also demonstrated reduced cell viability, mitochondrial dysfunction and activation of caspases 3 or 7 following treatment with Aβ_42_ oligomers in primary cortical [69] and hippocampal [70,71] neurons, HT-22 neuronal [72], SH-SY5Y [73,74], and LAN5 neuroblastoma cells in culture [43].

Using this in vitro AD model, we further examined potential neuroprotective actions of various DHEAS concentrations (10^−6^–10^−8^ M). Since 10^−7^ M DHEAS significantly counteracted an observed decline in the neuronal viability induced by toxic Aβ_42_ oligomers, we next investigated its effects on mitochondrial dysfunction, apoptosis and necrosis. The findings of Hoechst 33342 and propidium iodide staining revealed that 10^−7^ M DHEAS significantly decreased the apoptosis and to a much lesser extent the necrosis of neurons exposed to Aβ_42_ oligomers, suggesting dominant antiapoptotic effects of DHEAS. Moreover, administration of 10^−7^ M DHEAS significantly counteracted the decrease in cellular ATP levels, but not cell membrane integrity loss, both induced by Aβ_42_ oligomers, suggesting DHEAS effects on mitochondrial function but not on necrosis of neurons. In addition, we also observed that DHEAS blocked the increase in caspases 3 and 7 activity, as well as in the mRNA expression of *PI3K, Akt, Bax* and *Bcl2* genes (including *Bax/Bcl2* ratio), induced in primary neurons by treatment with Aβ_42_ oligomers. Together these findings suggest the involvement of mitochondria, caspases 3 and 7, as well as the PI3K/Akt and Bcl2 signaling network in the antiapoptotic properties of DHEAS in neurons. The neuroprotective effects of DHEA(S) against Aβ-toxicity were previously reported in cultured rat hippocampal neurons and hippocampal murine HT-22 cells [33,75]. Similar to our findings, some studies reported protective effects of DHEA(S) on mitochondrial membrane permeability and potential in primary chicken hepatocytes [76], Leydig cells [77] and human granulosa HO23 cells [78]. Goguadze et al. [79] also demonstrated that DHEAS attenuated an Aβ_1–42_-induced increase in mitochondrial ROS, commonly observed in AD pathology [79].

In line with our results, DHEA(S) also attenuated Aβ_25–35_ toxicity in B104 neuroblastoma cells [34] and mouse hippocampal neurons [22] by preventing the cells from entering both late apoptosis and necrosis via the activation of the PI3K-Akt signaling pathway [22]. The PI3K-Akt signaling pathway was found to regulate a variety of biological processes, such as proliferation, differentiation and apoptosis, and it has been recognized for its role in AD, as well as in neuroprotective and antiapoptotic effects of various agents [80]. In addition, DHEA(S) actions have been previously associated with the modulation of the Akt signaling pathway [24], as well as with the activation of prosurvival transcription factors CREB and NF-κB and downstream antiapoptotic Bcl-2 proteins [81,82].

Moreover, we further investigated the effects of DHEAS treatment on the Aβ plaque density in different brain regions of 3xTg-AD mice, an animal model of AD. The results of immunohistochemistry revealed the highest plaque count in the motor cortex (MC), followed by the basolateral amygdala (BLA), hippocampus (HC), and somatosensory cortex (SSC). These findings are in line with the reports demonstrating that individuals with dementia often exhibit poor motor function, including gait difficulties [83] and that impaired motor function may occur even before the onset of cognitive symptoms in both humans and animals [84,85]. However, although 3xTg-AD mice display cognitive and behavioral deficits that progress with age and perform poorly on some motor behavior tasks, they show enhanced performance on the rotarod, probably due to *P301L* transgene [86,87,88]. In our research, i.p. administration of 10 mg/kg DHEAS reduced the number of Aβ_43_ plaques in the MC but not in other brain regions (SSC, BLA, HC) of 3xTg-AD mice in a time-dependent manner. Specifically, the DHEAS-treated group exhibited a significant decrease in the number of Aβ_43_ plaques 48 h after treatment but not 24 h after treatment, indicating an effective treatment timeframe and suggesting that a longer period is needed for DHEAS beneficial actions. It is possible that DHEAS effects against Aβ accumulation are most prominent in MC of 6-month-old 3xTg-AD mice since in this region the highest number of plaques was observed. With the progression of AD pathology, the protective actions of DHEAS (especially if chronically applied) might also be significant in other brain areas of 3xTg-AD mice, especially since 3xTg-AD mice demonstrate an age-related decrease in DHEA(S) levels in the brain [89]. Chen et al. [90] demonstrated that weekly administration of another neurosteroid, allopregnanolone, for 6 months in 3xTg-AD male mice increased the survival of newly formed neurons and reduced Aβ burden in the hippocampus, cortex, and amygdala, and was particularly effective when given before the onset of pathological changes [90]. Another study also reported that DHEAS treatment in transgenic AD mice resulted in improved cognitive function and reduced Aβ accumulation [35]. In line with these findings are other studies demonstrating the neuroprotective effects of DHEA(S) against Aβ toxicity in mice [44,91,92]. In addition, synthetic enantiomers ent-PREGS and ent-DHEAS were also shown as effective in preventing Aβ_25–35_-induced memory deficits, when administered to male Swiss mice [34].

## 5. Conclusions

In conclusion, our findings obtained in vitro emphasize the complexity of AD pathology, particularly highlighting the toxic effects of Aβ_42_ oligomers in neurons. Moreover, our research demonstrated that DHEAS exerts neuroprotective and antiapoptotic actions against Aβ toxicity, mainly by preventing mitochondrial dysfunction and inhibiting caspase-3 and 7 activity, possibly via the PI3K/Akt and Bcl2 signaling network. Results obtained using 3xTg-AD mice as an animal model of AD revealed that DHEAS, 48 h after administration, significantly reduced the number of Aβ plaques in the MC, brain region with the highest plaque burden. Together, these findings suggest the neuroprotective effects of DHEAS against Aβ toxicity and accumulation in both cellular and animal models of AD. Therefore, DHEAS supplementation should be further studied as a novel preventive and/or therapeutic approach in AD [93].

## Data Availability

The original contributions presented in this study are included in the article/Supplementary Materials. Further inquiries can be directed to the corresponding author.

## Supplementary Materials

The following supporting information can be downloaded at: https://www.mdpi.com/article/10.3390/biomedicines13020432/s1, Figure S1. Primary mouse neurons after 5–7 days in vitro. Dendrites of neurons were stained with Anti-beta III Tubulin antibody (Abcam, Cambridge, UK) and Goat Anti-Rabbit IgG H&L (Alexa Fluor 488, Abcam, Cambridge, UK), whereas neuronal nuclei were stained with Hoechst 33342 (Thermo Fischer Scientific, Waltham, MA, USA) and detected with Olympus BX51 Fluorescence Microscope (Olympus, Tokyo, Japan). Figure S2. Image and size of (A) 10 μM Aβ_42_ monomers, (B) 10 μM Aβ_42_ oligomers, (C) 10 μM Aβ_42_ polymers/fibrils, detected by atomic force microscopy (AFM), and (D) SDS-PAGE electrophoresis. Figure S3. A representative sample of the cell viability profile obtained by treating primary mouse neurons with (A) vehicle (control) (B) 10 μM Aβ_42_ monomers, (B) 10 μM Aβ_42_ oligomers, (C) 10 μM Aβ_42_ polymers/fibrils, using MUSE Count & Viability reagent and MUSE™ cell analyzer (Luminex, Austin, TX, USA). Figure S4. Effects of 1 × 10^−7^ M DHEAS on apoptosis and necrosis of primary mouse neurons treated with 10 μM Aβ_42_ oligomers for 24 h determined by Hoechst 33342 (blue) and propidium iodide (red) staining and EVOS Cell Imaging Station (Thermo Fisher Scientific, Waltham, MA, USA). Table S1. The number Aβ plaques in different brain regions of 3xTg-AD mice, 24 and 48 h following vehicle (control) or 10 mg/kg DHEAS i. p. administration.
