# Supplementary material for: Neuroprotective Effects of Dehydroepiandrosterone Sulphate Against Aβ Toxicity and Accumulation in Cellular and Animal Model of Alzheimer’s Disease
Source: Biomedicines. 2025 Feb 11;13(2):432. doi: 10.3390/biomedicines13020432 (PMC11853520; doi:10.3390/biomedicines13020432)
Supplement: Supplementary file 1 [file biomedicines-13-00432-s001.zip › biomedicines-3454890-supplementary.pdf]

Supplementary Data

Article

Neuroprotective Effects of Dehydroepiandrosterone Sulphate Against Aβ Toxicity and Accumulation in Cellular and Animal Model of Alzheimer's Disease

Barbara Vuic, Tina Milos, Erika Kvak, Marcela Konjevod, Lucija Tudor, Szidónia Farkas, Gordana Nedic Erjavec, Matea Nikolac Perkovic, Dora Zelena, Dubravka Svob Strac

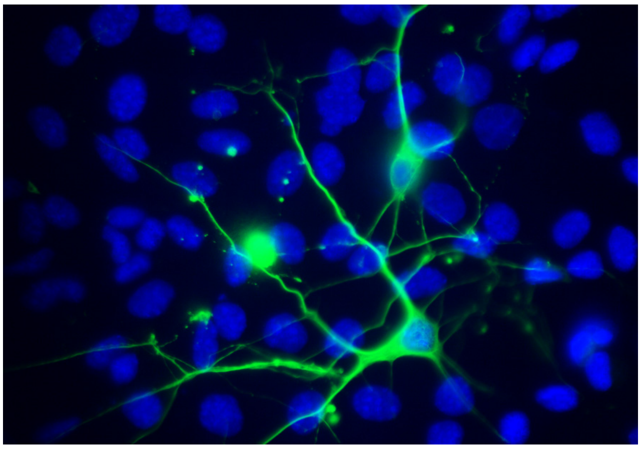

Supplementary Figure S1. Primary mouse neurons after 5-7 days *in vitro*. Dendrites of neurons were stained with Anti-beta III Tubulin antibody (Abcam, Cambridge, UK) and Goat Anti-Rabbit IgG H&L (Alexa Fluor 488, Abcam, Cambridge, UK) , whereas neuronal nuclei were stained with Hoechst 33342 (Thermo Fischer Scientific, Waltham, MA, USA) and detected with Olympus BX51 Fluorescence Microscope (Olympus, Tokyo, Japan).

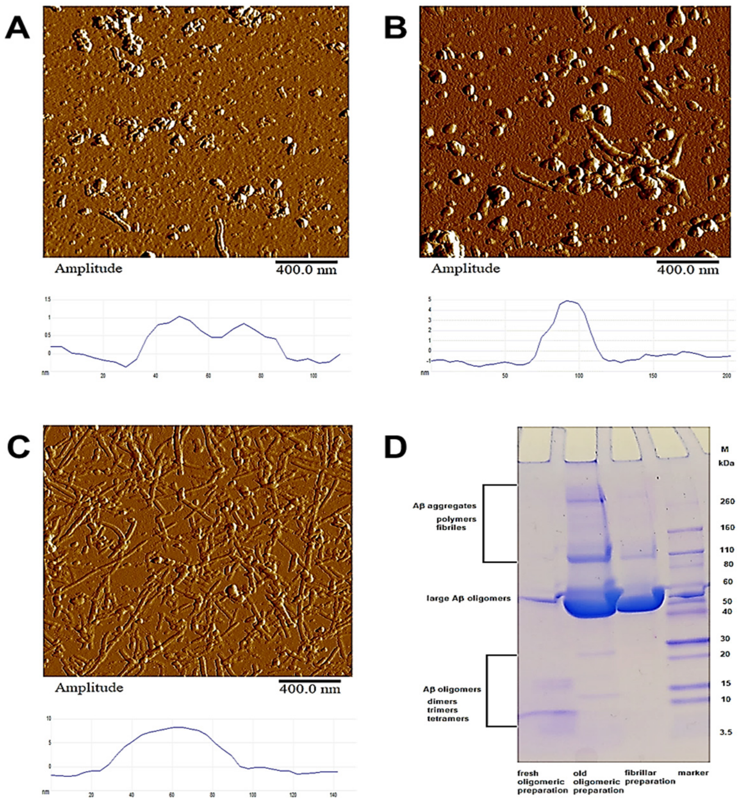

Supplementary Figure S2. Image and size of A) 10 µM Aβ42 monomers, B) 10 µM Aβ42 oligomers, C) 10 µM Aβ42 polymers/fibrils, detected by atomic force microscopy (AFM), and D) SDS-PAGE electrophoresis.

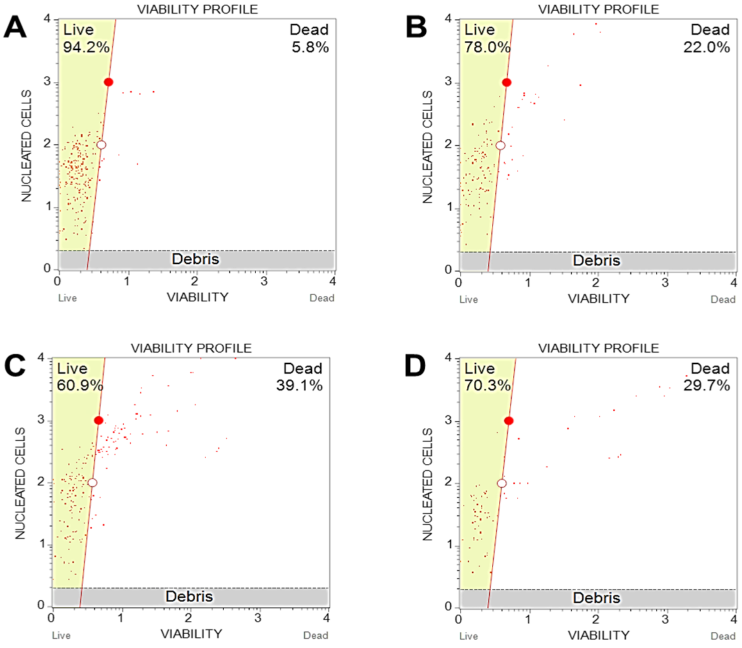

Supplementary Figure S3. A representative sample of the cell viability profile obtained by treating primary mouse neurons with A) vehicle (control) B) 10 µM Aβ42 monomers, B) 10 µM Aβ42 oligomers, C) 10 µM Aβ42 polymers/fibrils, using MUSE Count & Viability reagent and MUSE™ cell analyzer (Luminex, Austin, TX, USA).

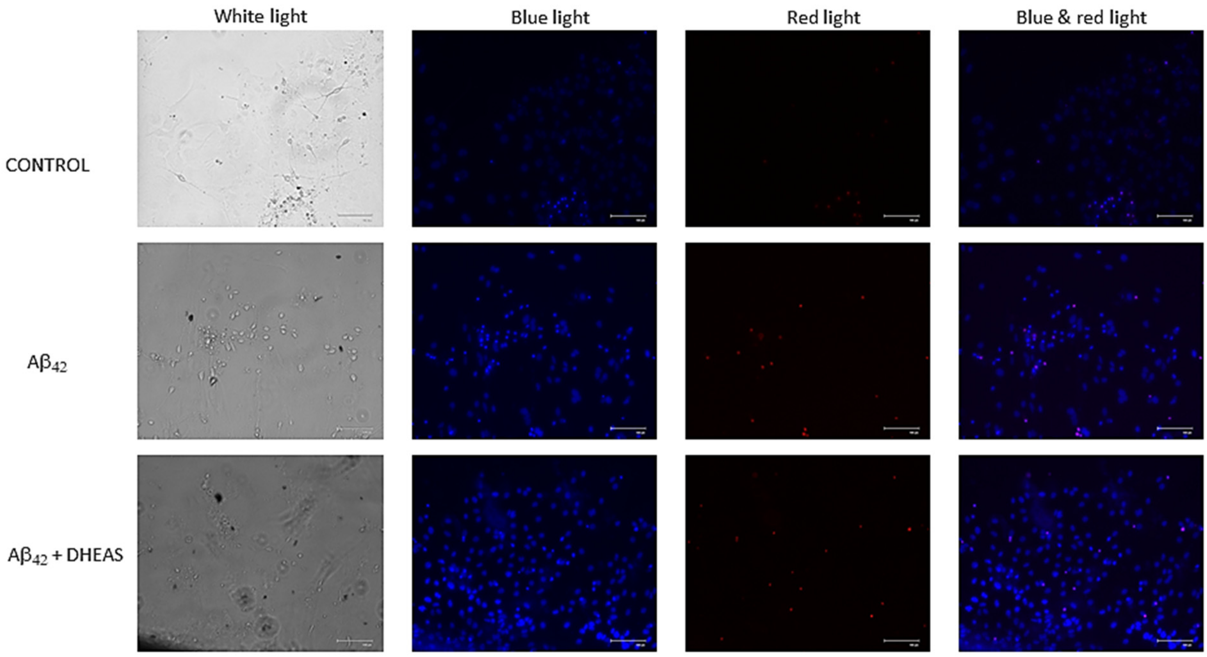

Supplementary Figure S4. Effects of 1 x 10^{-7} M DHEAS on apoptosis and necrosis of primary mouse neurons treated with 10 μM $A\beta_{42}$ oligomers for 24 h determined by Hoechst 33342 (blue) and propidium iodide (red) staining and EVOS Cell Imaging Station (Thermo Fisher Scientific, Waltham, MA, USA).

Supplementary Table S1. The number Aβ plaques in different brain regions of 3xTg-AD mice, 24 and 48 h following vehicle (control) or 10 mg/kg DHEAS i. p. administration.

						TWO-Way ANOVA		
**Brain region**	**Animal group**	**Timepoint**	**Mean (no. of plaques)**	**Mean normalised**	**SEM**	**Interaction**	**24h x 48 h**	**Control x DHEAS**
**MC**	Control	24h	153,8472	1,0104	0,1184	F(1, 20) = 5.5430<br>P=0.0289	F(1, 20) = 4.5050<br>P=0.0465	F(1, 20) = 14.2200<br>P=0.0012
	DHEAS	24h	128,3333	0,8467	0,1151			
	Control	48h	159,9722	1,0372	0,1578			
	DHEAS	48h	60,8833	0,6733	0,0364			
**SSC**	Control	24h	41,0750	1,0663	0,3367	F(1, 18) = 2.9260<br>P=0.1044	F(1, 18) = 3.1690<br>P=0.0919	F(1, 18) = 0.1846<br>P=0.6725
	DHEAS	24h	44,7188	1,4586	0,1841			
	Control	48h	60,2119	1,0534	0,1000			
	DHEAS	48h	41,2250	0,8187	0,2994			
**BLA**	Control	24h	128,7250	1,0217	0,0562	F(1, 23) = 0.0668<br>P=0.7983	F(1, 23) = 5.674e-006<br>P=0.9981	F(1, 23) = 0.2697<br>P=0.6085
	DHEAS	24h	132,5333	0,9179	0,1836			
	Control	48h	98,6094	0,9869	0,1479			
	DHEAS	48h	98,1706	0,9521	0,1093			
**HC**	Control	24h	69,1733	1,0169	0,0853	F(1, 22) = 0.0011<br>P=0.9730	F(1, 22) = 0.4442<br>P=0.5120	F (1, 22) = 1.8560<br>P=0.1869
	DHEAS	24h	81,3417	1,1827	0,1419			
	Control	48h	46,6319	0,9295	0,1477			

MC - Motor cortex, SSC - Somatosensory cortex, BLA - Basolateral amygdala, HC –Hippocampus. Results are expressed as means ± SD from 6 mice per group and analyzed by two-way ANOVA followed by Tukey's multiple comparisons test. Significant results are shown in red color.
